# XRD and TG-DTA Study of New Alkali Activated Materials Based on Fly Ash with Sand and Glass Powder

**DOI:** 10.3390/ma13020343

**Published:** 2020-01-11

**Authors:** Dumitru Doru Burduhos Nergis, Mohd Mustafa Al Bakri Abdullah, Andrei Victor Sandu, Petrică Vizureanu

**Affiliations:** 1 Faculty of Materials Science and Engineering, “Gheorghe Asachi” Technical University, Blvd. D. Mangeron 71, 700050 Iasi, Romania; bunduc.doru@yahoo.com (D.D.B.N.); mustafaalbakri79@gmail.com (M.M.A.B.A.); 2 Romanian Inventors Forum, Str. Sf. P. Movila 3, 700089 Iasi, Romania; 3 National Institute for Research and Development in Environmental Protection, 294 Splaiul Independenței Blv, 060031 Bucharest, Romania

**Keywords:** geopolymers, fly ash, thermal behavior, Thermogravimetry-Differential Thermal Analysis (TG-DTA), XRD

## Abstract

In this paper, the effect on thermal behavior and compounds mineralogy of replacing different percentages of fly ash with compact particles was studied. A total of 30% of fly ash was replaced with mass powder glass (PG), 70% with mass natural aggregates (S), and 85% with mass PG and S. According to this study, the obtained fly ash based geopolymer exhibits a 20% mass loss in the 25–300 °C temperature range due to the free or physically bound water removal. However, the mass loss is closely related to the particle percentage. Multiple endothermic peaks exhibit the dihydroxylation of β-FeOOH (goethite) at close to 320 °C, the Ca(OH)_2_ (Portlandite) transformation to CaCO_3_ (calcite) occurs at close to 490 °C, and Al(OH)_3_ decomposition occurs at close to 570 °C. Moreover, above 600 °C, the curves show only very small peaks which may correspond to Ti or Mg hydroxides decomposition. Also, the X-ray diffraction (XRD) pattern confirms the presence of sodalite after fly ash alkaline activation, whose content highly depends on the compact particles percentage. These results highlight the thermal stability of geopolymers in the 25–1000 °C temperature range through the use of thermogravimetric analysis, differential thermal analysis, and XRD.

## 1. Introduction

In recent years, strong technological development, the population increase, and the rapid development of the house-building industry in particular have led not only to a large lack of housing areas but also to high demand for building materials. The use of waste resulting from coal combustion in power plants offers two major advantages for this purpose: first, large tailings areas can be liberated by utilizing the waste, and second, a soil contaminant material can be converted into an advanced material with appropriate chemical and mechanical properties for engineering application through a geopolymerization process [1]. Geopolymers are inorganic materials, based on silica-alumina, which are chemically balanced by Group I oxides [2]. These are rigid gels, created under normal conditions of temperature and pressure, which can then be transformed into crystalline or glass-ceramic materials that are similar to zeolite materials [3]. A geopolymer, resulting from the exothermic process involving oligomers, is a very long reticular polymer with silicon groups (SiO_4_) and a specific tetragonal network of aluminum oxide (AlO_4_) [4]. The bonds between these tetragons are balanced by alkaline ions of K^+^, Na^+^, or Li^+^ [4]. Any geopolymer can be divided into two main constituents: the base material and the activator (an alkaline liquid) [5]. The major constituent is the base material, which must be rich in silicon and aluminum and can be a natural mineral, such as clays, kaolin, etc. or alternatively can be a form of waste, such as fly ash, red mud, slag, etc. [1].

Due to their physical [6], mechanical [7], and chemical properties [8,9], geopolymers show high usefulness in multiple civil engineering applications [10,11] as a replacement material for conventional cement or ceramics [12,13,14,15]. Therefore, the thermal behavior and phase transition of fly ash based geopolymers during the exposed temperature range must be analyzed in order to evaluate the stability of their structure.

An additional advantage is the fact that the geopolymer microstructure contains multiple unreacted particles, which are continuously reacting with the extra-gel remained in the micropores [16,17]. As a result, some harmful cracks and pores could be repaired through the self-healing mechanism [18,19]. Obviously, this self-healing feature positively influences the time depending behavior of the composites due to its high durability. Despite the fact that geopolymers possess many chemical and mechanical properties and can be obtained through simple methods, most of them are obtained from natural minerals instead [20]. Therefore, it is essential to design, create, and characterize new geopolymers that use mineral waste as a source of raw materials, especially indigenous waste, and recyclized reinforcing particles. This is encouraged for both economic and environmental reasons, because through the geopolymerization reaction, we can obtain useful materials using “free” wastes that have negative effects on dumping areas [21].

There are multiple studies regarding the influence of different types of particles on the mechanical properties of geopolymers [17,22,23,24,25,26]. However, the presence of these particles will influence all the characteristics of the geopolymers, including their thermal behavior. The aim of this study is to evaluate the thermal behavior changes and the phase transition due to the introduction of different types of particles in new geopolymers based on indigenous fly ash.

## 2. Materials and Methods

Geopolymerization is a multiple-stage chemical reaction which occurs when a raw material rich in aluminum and silicon oxides is mixed with an alkaline solution of sodium silicate and sodium hydroxide [27,28]. Also, this reaction is mainly influenced by the raw material characteristics [29], activator concentration, and curing process (drying time and temperature) [30]. In this study, fly ash was used as the main raw material, and different percentages of glass powder and/or natural aggregates were introduced in the binder as reinforcement particles. Their chemical composition was analyzed by using X-ray fluorescence (XRF) involving XRF S8 Tiger equipment (Bruker, Karlsruhe, Germany).

### 2.1. Materials

Geopolymers consist of two main components: the liquid component (activator solution) and the solid component (the material rich in aluminum and silicon oxides and the reinforcing particles).

#### 2.1.1. Indigenous Fly Ash

**Figure 1:**
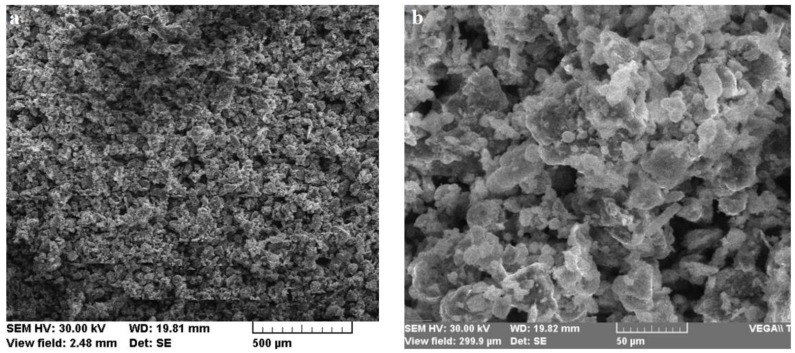
Scanning Electron Microscope (SEM) micrographs of indigenous fly ash after sifting: (**a**) 100X magnification; (**b**) 750X magnification.

Fly ashes are, generally, solid torque spheres which result from coal combustion in power plants burning chambers [31]. This micrometric powder ends up being deposited in huge areas near many cities all over the world. Because different dumps present different chemical compositions, the activation solution, as well as the ratio between constituents, must be calculated. The performance of fly ash in geopolymers is strongly influenced by its physical, chemical, and mineralogical properties, and moreover, by its particle dimensions. While the mineralogical and chemical composition (Table 1) depends mainly on the coal composition, the particles can be ground or sifted (Figure 1).

In Romania, there are large areas covered by industrial waste from coal burning in the city’s power plants. The indigenous thermal power plant ash used for the geopolymer tests comes from CET II—Holboca Iasi Romania ash dumps, which occupied an area of approximately 50 hectares in 2013 [32].

According to the Standard ASTM C618-92a, indigenous fly ash belongs to class F because it has a main oxides (silicon, aluminum and iron) sum that is higher than 70% (Equation (1)):

SiO_2_ + Al_2_O_3_ + Fe_2_O_3_ = 47.8% + 28.6% + 10.2% = 86.6%. (1)

#### 2.1.2. Glass Powder

Another waste that appears in large quantities due to industrialization is glass. This inert material does not decompose naturally, producing negative effects on the environment following storage in landfills. Therefore, the use of glass particles in the manufacturing of environmentally friendly materials has become a worldwide concern. Due to the incorporation ability of geopolymer paste, the introduction of glass powder into the composition of these materials can be done using simple methods.

**Figure 2:**
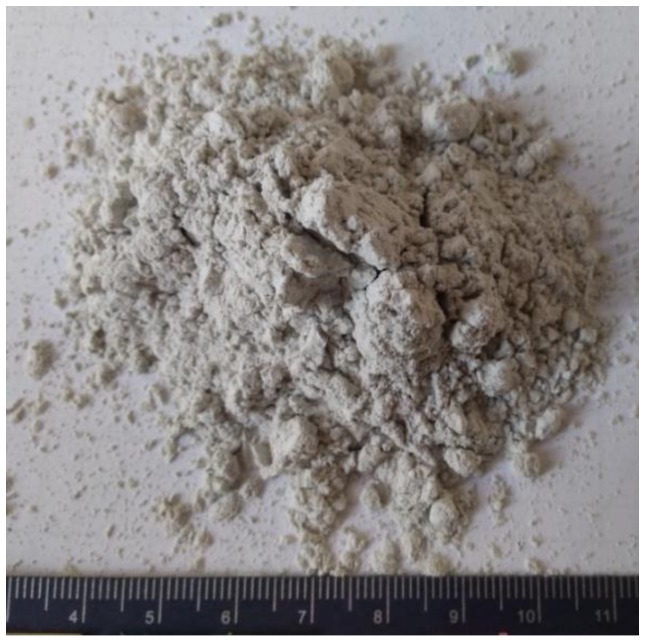
Glass powder.

The glass powder (Figure 2) used as a reinforcing element in geopolymer samples contains only particles smaller than 100 µm (SR EN 933-1/2012) and is obtained by conducting glass waste grinding in a local factory.

By comparing the chemical composition (Table 2) with that of the thermal power plant ash (Table 1), the glass powder contained much higher SiO_2_, CaO, and Na_2_O, but much lower Al_2_O_3_. However, according to several studies [33], glass powder reacts in alkaline environments. Therefore, this should increase the geopolymerization rate.

By using glass particles for geopolymers manufacturing, two main advantages emerge: the first is related to waste recycling and second refers to improving mechanical properties by introducing particles with high mechanical properties.

#### 2.1.3. Natural Aggregates

In order to improve the mechanical characteristics of geopolymers based on local powerplant ash, different quantities or types of aggregates can be added to the composition. Besides the use of waste, another category of reinforcing elements studied [25,34] worldwide is natural aggregates (sand). Depending on the geometric peculiarities of the particles, by introducing them in the geopolymer matrix, compressive strength increases of up to 150% can be obtained. The quantity and type of aggregate used is chosen according to the particle size distribution of the sand, because it may affect the homogeneity of the samples, but also their porosity.

The sand granulometric characteristics analysis conducted by using sifting (SR EN 933-1/2012) was performed after drying the aggregates, in order to reduce the measurement errors due to the fine particles sticking or adhesion to the sieve surface. According to the particle size distribution, close to 30% of particles had a diameter higher than 1.25 mm, and 50% (d50) had a diameter lower or equal to 0.19 mm. Therefore, the type of sand used belongs to the 0/4 aggregate class because all particles pass through the 4 mm mesh sieve (SR ISO 3310-3). The XRF analysis of sand indicated the following composition: 98.8% SiO_2_, 0.57% Al_2_O_3_, 0.33% Fe_x_O_y,_ and the rest being CaO, Na_2_O, and other materials as traces.

#### 2.1.4. Sodium Silicate

Sodium silicate is made by a sand (SiO_2_) fusion with sodium or potassium carbonate (Na_2_CO_3_ or K_2_CO_3_) at temperatures above 1100 °C and dissolving the high-pressure vapor product in a semi-viscous liquid known as silicate. Silicate is rarely used as an independent activator because it does not have a sufficient activation capacity to initiate a geopolymerization reaction.

A commercially purchased high purity Na_2_SiO_3_ solution (Scharlab S.L., Barcelona, Spain) with a density of 1.37 g/cm^3^ and a lower pH than 11.5 was used in this study.

#### 2.1.5. Sodium Hydroxide

The NaOH solution concentration and molarity strongly influence the final properties of the geopolymers. The high concentrations of the NaOH solution result in high resistance to the early reaction stages. NaOH-activated geopolymers possess high crystallinity, having better stability in acidic or sulfate environments [35].

The NaOH solution was prepared at a 10-molar concentration by dissolving the high purity (99%) NaOH flakes in distilled water for 24 h before use (mixing).

#### 2.1.6. Sample Preparation

**Figure 3:**
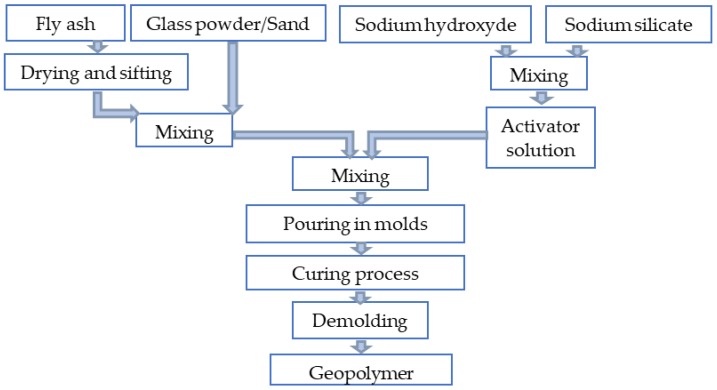
Process flow diagram.

The samples mixture was prepared according to the BS EN 196-1:1995 by means of a variable speed mixer. In order to increase the homogeneity of the samples, firstly, the solid component was poured into the mixer and stirred in a dry state for 4 min. Secondly, the liquid component was added gradually and mixed for 10 min until a homogeneous binder was obtained. The mix proportion of liquid and solid component of each sample are presented in Table 3, and the process flow diagram is shown in Figure 3.

Because the geopolymer characteristics and properties depend on multiple factors, it is essential to set the optimal parameters to be specific to the raw material, the activation solution, and the curing process. Therefore, the following parameters were used in this study:

- a raw materials relative humidity of 0;
- fly ash particles lower than 80 µm;
- glass powder particles lower than 100 µm;
- sand particles lower than 4 mm;
- curing temperature of 70 °C;
- curing time of 8 h.

During the geopolymerization process, minerals rich in aluminum and silicon pass through several phases. In the first phase, these are dissolved by the alkaline solution, forming a gel whose viscosity is given by the ratio between solid and liquid (Equation (2)). In the second phase, the reorganization of the molecules takes place, while water is removed and the material hardening begins.

(2) $$\frac{g\;\mathrm{of}\;\mathrm{solid}\;\left(\mathrm{powerplant}\;\mathrm{ash}\right)}{g\;\mathrm{of}\;\mathrm{activating}\;\mathrm{solution}\;\left(\mathrm{sodium}\;\mathrm{silicate}+\mathrm{sodium}\;\mathrm{hydroxide}\right)}=1.5$$

In order to evaluate the effect on thermal behavior and compounds mineralogy of replacing different percentages of fly ash with reinforcing elements, four types of geopolymers samples were obtained and studied.

### 2.2. Methods

Simultaneous thermal analysis consisting of thermogravimetric analysis (TGA) and Differential thermal analysis (DTA) was performed on the obtained samples in order to evaluate their thermal behavior. Because the DTA curve showed multiple peaks in the temperature range where the evaluation was made, X-ray diffraction (XRD) was performed to confirm the phase transition during heating.

#### 2.2.1. Simultaneous Thermal Analysis

The sample’s mass evolution by TGA was analyzed simultaneously with the phase transformations analysis using DTA by means of a STA PT-1600 equipment (Linseis, Selb, Germany). The analysis was performed in the 25–1000 °C temperature range, with a heating rate of 10 °C/min on samples and a mass lower than 50 mg, in a static air atmosphere.

Materials analysis conducted using TG-DTA emphasized their thermal stability and the content/type of volatile compounds through two curves simultaneously plotted based on the temperature.

#### 2.2.2. X-ray Diffraction

X-ray diffraction (XRD) is a technique used to identify crystalline phases in different materials and for quantitative analysis of these phases. XRD is used, in particular, due to the superior highlighting of the three-dimensional atomic structure that directly influences the properties and characteristics of the materials. In order to analyze the mineralogical composition of the obtained geopolymers, an X’Pert Pro MPD equipment (Malvern Panalytical Ltd., Eindhoven, The Netherlands) equipped with a copper x-ray tube and a single channel detector was used. The diffractograms between the X-ray intensity on the ordinate and the Bragg angle, θ, on the abscissa were performed on a θ–2θ angle range between 5° and 90° through continuous scanning at a step size of 0.013° at every 60 s, with a scan speed of 0.054 (°/s) at a 45 KV voltage and 40 mA current intensity.

The mineralogical changes produced by the alkaline activator on the fly ash was analyzed on powder obtained by grinding the samples maintained in normal atmosphere conditions (clean air, ≤20 °C) for 90 days, and after being analyzed by TG-DTA.

## 3. Results and Discussion

### 3.1. Thermal Behavior Evaluation

The TG-DTA simultaneous thermal analysis was used to evaluate the thermal stability of geopolymers after replacing high percentages of fly ash with two types of particles. By monitoring the mass change during the heating of samples, the fraction of volatile compounds could be determined, so if the DTA curve is plotted at the same time, the mass change at specific temperatures could confirm the quantity of a specific compound.

The DTA curves of samples show multiple peaks at 123–130 °C, 185 °C, 232–240 °C, 312–358 °C, 490–497 °C, and 572–576 °C, respectively. These peaks correspond to the removal of water molecules, which are free or bound are with the structural compounds. In totally inorganic materials, such as geopolymers, water can be found in two main forms:

(i) hygroscopic (free) water, which is removed at rising temperatures up to 120 °C [36]. This water is absorbed into the structure due to the hygroscopicity of geopolymers [37].

(ii) strong physically bonded water which is removed in the 120–300 °C temperature range. This type of water can be divided into three sub-types:

- crystallization water (anionic and cationic or coordinative) which is removed from the structure in the 120–200 °C temperature range. This sub-type of water molecules are bonded in the structure during the formation of crystals from aqueous solution [38].
- water from hydrogels that can be intercrystalline and network types that interact with the crystallization water. This sub-type of water is removed during heating in the 180–300 °C temperature range [39].
- zeolitic water from cavities and channels, which is removed from the structure in the 200–300 °C temperature range [37,40].

When the temperatures exceed 300 °C, the (iii) chemically bound water starts being removed. The peaks on the DTA curve above this temperature corresponded to the decomposition of M (metal) and OH groups compounds [39,41,42]. These compounds exist in the fly-ash based geopolymers structure in different forms, such as:

- Acids: M-O^−^H^+^ (Si(IV), Ti(IV), Fe(III))
- Basics: M^+^HO^−^ hydroxide (Na, Ca (II), K, Mg (II))
- Neutral: M-OH hydroxyl (Al (III), Mn (III)).

**Figure 4:**
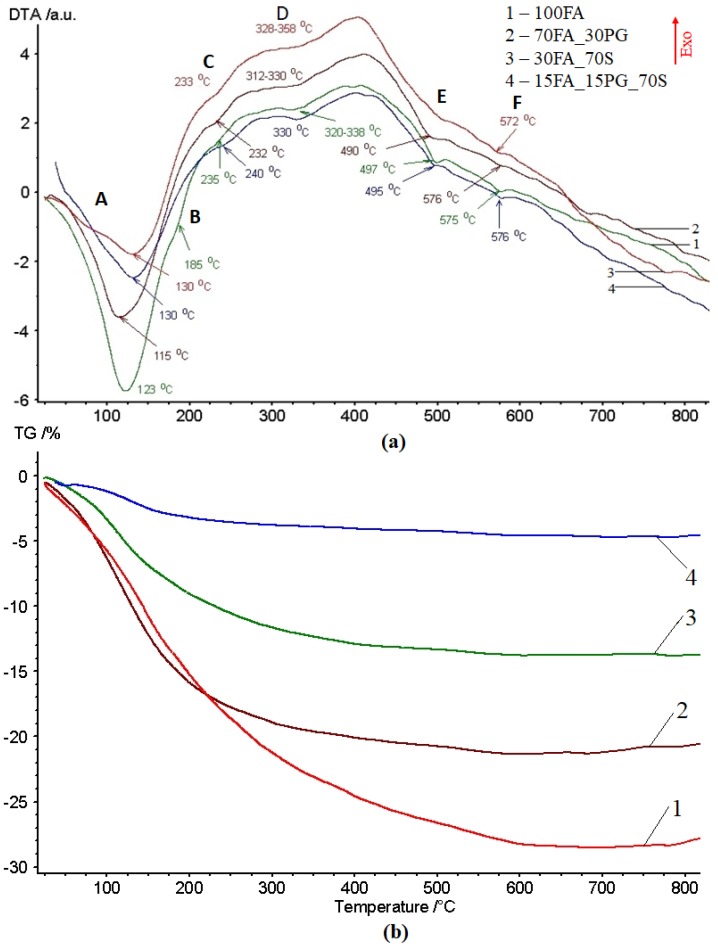
TG-DTA curves in the 22–820 °C temperature range: (**a**) DTA curves; (**b**) TG curves.

**Figure 5:**
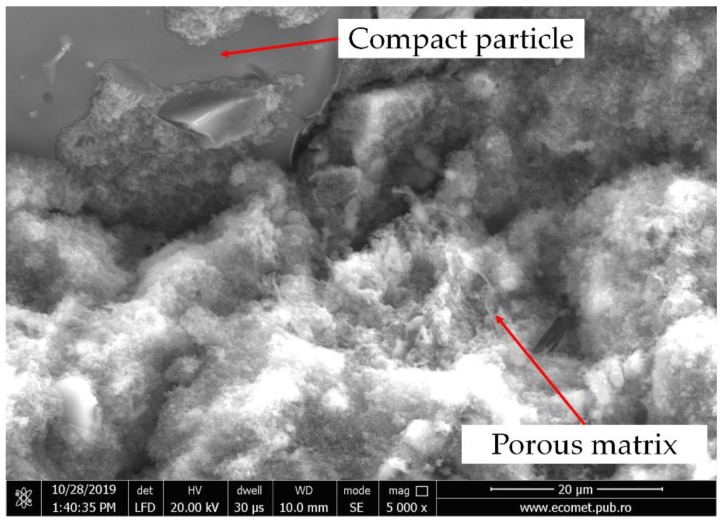
SEM micrographs of geopolymers with particles.

The DTA curves (Figure 4a) of the analyzed samples showed an endothermic peak whose minimum was positioned at 123 °C for an 100FA sample, 115 °C for an 70FA_30PG sample, and 130 °C for 30FA_70S and 15FA_15PG_70S, respectively. The peak “A” corresponds to the overlapping of the removing of hygroscopic water evaporation and crystallization water removal [37]. By comparing the peaks broadening, it can be seen that by increasing the percentage of compact particles, the amount of water in these forms is lower. Because the used particles are compact bodies (Figure 5), the porosity of the sample can be related only with the percentage of fly ash. Therefore, high fly ash content ensures a highly porous structure which will increase the amount of absorbed water.

The “B” peaks which are in the temperature range of hydrogel water removal are higher in the case of the 100FA sample. This can be related to the hydrogel-forming capability of fly ash during geopolymerization [43].

Close to 230 °C, another peak, “C”, appeared. During this endothermic reaction, the water molecules were removed from the calcium silicate hydrate (C-S-H), C-S-H with Al in its structure (C-A-S-H), and sodium aluminosilicate hydrate (N-A-S-H) channels and pores [44,45].

The “D” peaks corresponded to the iron oxides transition from FeO(OH) amorphous phase (Goethite) into the α-Fe_2_O_3_ (Hematite) crystalline phase (Equation (3)) [46,47,48,49]. The transformation reaction of Fe compounds occurred at around 300 °C but could be moved to higher temperatures due to the presence of silica and aluminum [50].

The “E” peaks represented an endothermic reaction in the 490–497 °C temperature range and corresponded to calcium hydroxide Ca(OH)_2_ (Portlandite) decomposition following a reaction with carbon from the atmosphere, resulting in CaCO_3_ and H_2_ (Equation (4)) [51,52,53].

Also, at up to 570 °C, the “F” peaks which appeared on the DTA curve corresponded to the α-quartz to β-quartz conversion and the reaction between the unreacted particles and the activator caught in gel pores [54]. However, in the same temperature range, aluminum hydroxide, (Al(OH)_3_) decomposition occured (Equation (5)) [55,56,57,58].

FeO(OH)(s) + 3H^+^(aq) = Fe^2+^(aq) + 2H_2_O(aq) (3)

Ca(OH)_2_(s) + CO(g) → CaCO_3_(s) + H_2_(aq) (4)

2Al(OH)_3_(s) → Al_2_O_3_(s) + 3H_2_O(aq) (5)

In addition, in the same temperature range, the water resulting from the silicon or aluminum hydroxide groups condensation could appear. According to references [5,59], this chemical reaction consists of (Equation (6)):

≡M-OH + HO-M≡ → ≡M-O-M≡ + H_2_O (6)

**Figure 6:**
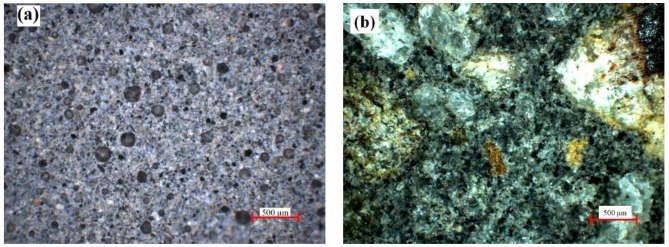
Optical micrographs of: (**a**) sample 100FA; (**b**) sample 30FA_70S.

As can be seen in Figure 6a, the obtained geopolymers presented large pores distributed on the entire analyzed surface. After introducing the aggregates, the large pores especially decreased in number (Figure 6b).

The samples mass loss (Figure 4b) caused by hygroscopic water evaporation was close to 10% for the 100FA sample, 8% for 70FA_30PG, 5% for 30FA_70S, and 2% for 15FA_15PG_70S, respectively. The water absorbance capacity of samples was related to the calcium oxides and silica gel concentration. Up to the temperature when the hydrogels water is removed, the samples mass decreased to close to 16% in the case of 100FA and 70FA_30PG samples, while the samples with sand show lower than 10% mass reduction. However, up to 250 °C, the mass decreases reached close to 18% in the case of 100FA and 70FA_30PG samples, 12% in the case of 30FA_70S, and only 3% in the case of 15FA_15PG_70S.

Even if the percentage of mass loss up to this temperature is relatively high, because these types of water molecules are free or physically bonded, their influence on the mechanical properties is insignificant. However, if these materials are subjected to freeze-thaw cycles, cracks formation may occur due to the water (ice) from the expansion of the pores, which reduces the mechanical resistance of the geopolymers [60].

In the 360–700 °C temperature range, the mass loss is due to the removing of chemical bound water molecules. Therefore, an increase of between 460 °C and 515 °C corresponds to a 1% mass reduction of the 100FA sample and close to a 0.2% mass reduction of 15FA_15PG_70S sample, which is related to the CaOH decomposition. Also, in the Al(OH)_3_ and FeO(OH) decomposition temperature range, the samples mass loss are lower than 1%.

Furthermore, above this temperature range, the DTA curves still show small peaks. These endothermic or exothermic reactions correspond to the decomposition of CaCO_3_ at close to 750 °C [61] (Equation (7)), Ti(OH)_4_ close to 790 °C [62], or Mg(OH)_2_ close to 670 °C [63]. Yet, these compounds exist only at the tracks level. Therefore, the effects on sample characteristics are low. At over 700 °C, a mass gain can be observed, which appears to be due to the oxidation of oxygen-poor iron species or pure iron [64].

CaCO_3_(s) → CaO(s) + CO_2_(g) (7)

The 100FA sample shows four peaks with the largest area. Therefore, the compounds that decompose in the analyzed temperature range come from the power plant fly ash particles.

### 3.2. Mineralogical Evaluation

The initial phases specific to the raw material and the transition in other phases that is specific to the zeolites are governed by the characteristics of the geopolymerization reaction. This transition is based on the raw material dissolution under alkaline conditions, resulting in reactive precursors of Si(OH)_4_ and Al(OH)_4_, and the polymerization and precipitation of the system, resulting in condensation of Si-O-Al molecules in various compounds.

**Figure 7:**
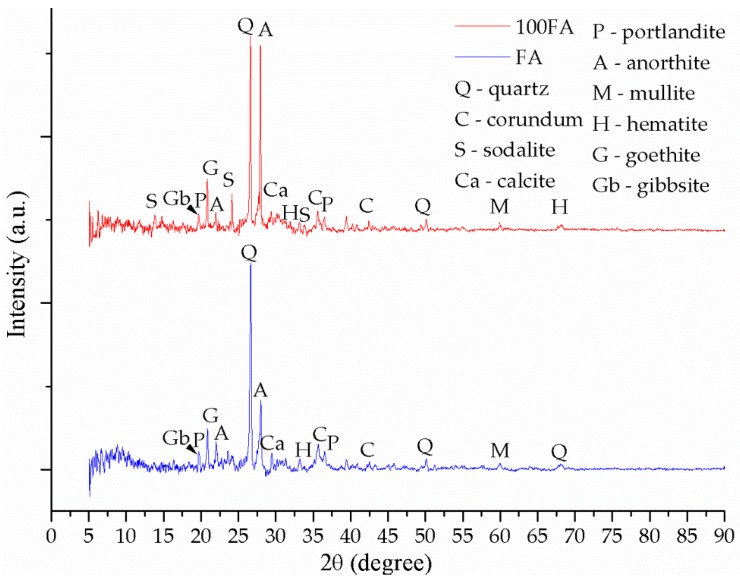
XRD patterns of fly ash powder and sample 100FA. (the Gb and P peaks with the highest intensity are overlapping).

The fly ash diffractogram (Figure 7) shows multiple peaks specific to the main chemical components oxides, such as Q—quartz, C—corundum, M—mullite or H—hematite, but also other more complex crystalline phases including calcium, titanium, or magnesium, such as A—anorthite, G—goethite, Al—albite, Ca—calcite, P—portlandite, Gb—gibbsite, magnesium hydroxide, etc. Both in the case of the raw material diffractogram and in the case of the obtained geopolymers, most of the peaks were positioned between 20° and 45° (2θ). Moreover, the peaks with the highest intensities specific to the quartz and corundum were positioned between 25° and 30° (2θ).

The detected quartz or silicon dioxide is a mineral with a tetrahedral structure formed between silicon atoms and oxygen, crystallizing in the hexagonal system. Its concentration positively influences the mechanical properties of geopolymers, due to the quartz particles capacity of creating barriers for crack propagation.

The corundum detected crystallizes in the rhombohedral system, which is known as one of the main aluminum oxides. This compound is essential for geopolymers due to its hardness being close to that of diamond.

Mullite crystallizes in the orthorhombic system, and is a less commonly encountered compound that forms between aluminum, silicon, and oxygen. Due to its very high melting temperature, 1840 °C, the presence of this mineral produces the increase of the geopolymers refractivity.

Hematite crystallizes in the rhombohedral system due to being a compound of iron with oxygen. It has the same crystallographic structure as that of the corundum, being frequently encountered with it.

Augite crystallizes in the monoclinic system being a complex compound of calcium, magnesium, silica and oxygen. It has a stone-like structure and color, and is rarely encountered with a shiny surface.

The anorthite crystallizes in the triclinic (anorthic) crystalline system, as it has the richest calcium content in the group of plagioclase feldspars. It is found in several colors and consists of calcium, aluminum, silicon, oxygen, but also potassium, sodium, iron, and titanium at trace levels.

Sodalite crystallizes in the cubic system as a mineral complex formed by the reaction between sodium and chlorine with the main elements of the raw material (aluminum, silicon, and oxygen). The natural sodalite consists of an Al-O-Si network that encompasses Cl^+^ cations, but the one resulting from geopolymerization shows inter-structural Na^+^ cations, similar to zeolites [3].

Following the geopolymerization chemical reaction between the fly ash and the activation solution, the main phase specific to the raw material, the quartz, whose peak is positioned at 26.62°, 2θ, decreases in intensity as a result of the decrease of the glass phase, but there is a significant increase in the anorthite intensity, 28.03°, 2θ, while new peaks specific to the phases created as a result of the reaction between Na and the other compounds also appear.

The diffractogram specific to the sample 100FA (Figure 7) shows the formation of the most important phase specific to the geopolymerization, i.e., sodalite, which shows three peaks between 8° and 35°, with the highest intensity at 24.50°, 2θ. The appearance of such a phase specific to zeolites suggests the formation of a mesoporous material (contains small pores with a diameter between 20 and 50 nm) of semi-crystalline nature [65]. The sodalite content formed is directly proportional to the cation exchange capacity between the raw material and the activation solution. However, prior to and after activation, secondary phases, such as corundum with the highest intensity peak at 35.47°, 2θ, portlandite with the highest intensity peak at 36.48°, 2θ, mullite with the highest intensity peak at 60.76°, 2θ, hematite with the highest intensity peak at 33.69°, 2θ, goethite with the highest intensity peak at 21.09°, 2θ, and calcite were confirmed.

**Figure 8:**
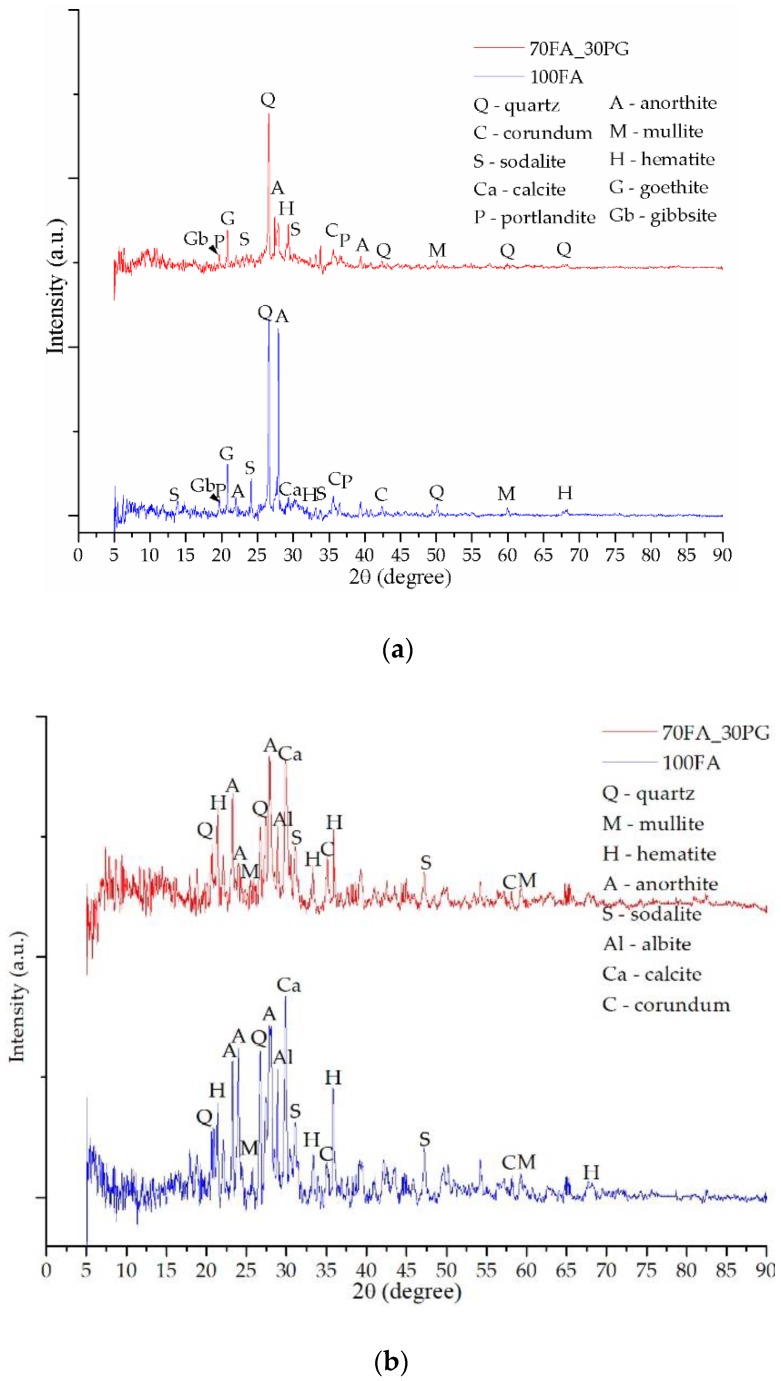
XRD patterns of sample 100FA and sample 70FA_30PG: (**a**) prior TG-DTA analysis; (**b**) after TG-DTA analysis.

After replacing 30% of the fly ash quantity with glass particles, the sample 70FA_30PG results (Figure 8a) showed a decrease in intensity of the phases characteristic of the chemical reaction between the activation solution and ash.

**Figure 9:**
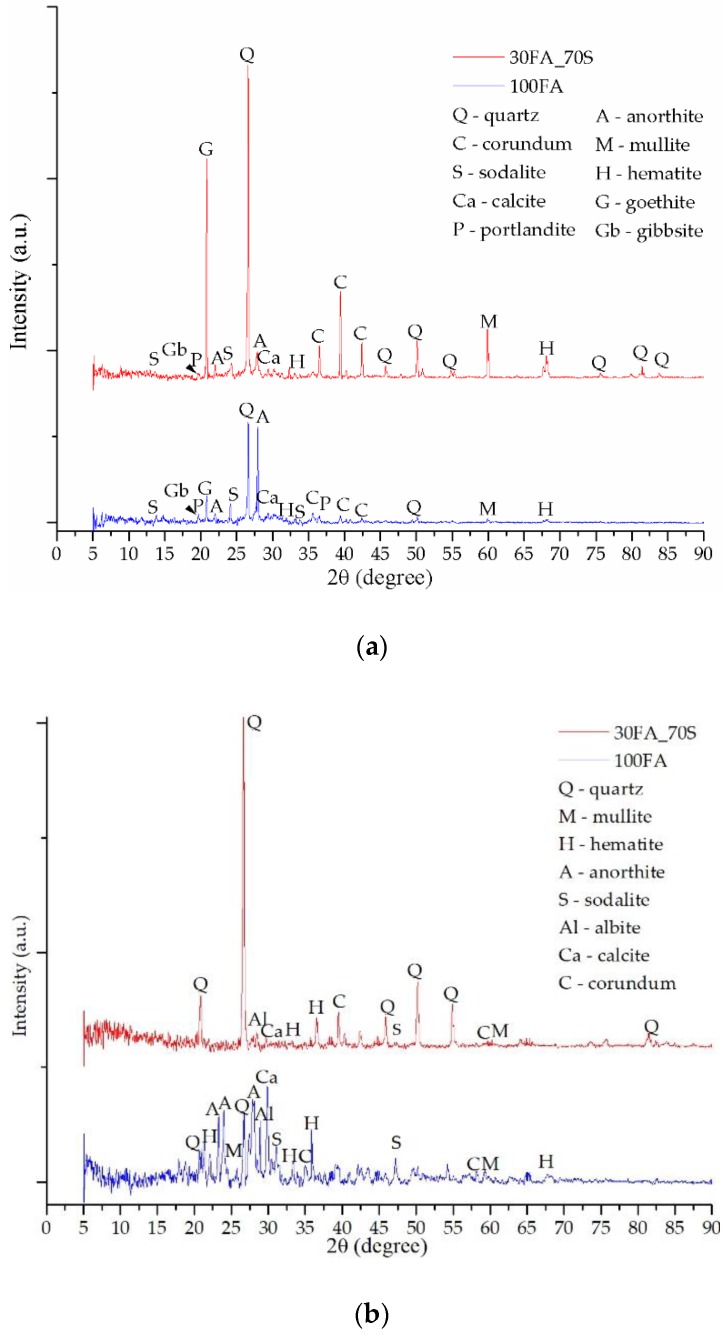
XRD patterns of sample 100FA and sample 30FA_70S: (**a**) prior TG-DTA analysis; (**b**) after TG-DTA analysis.

**Figure 10:**
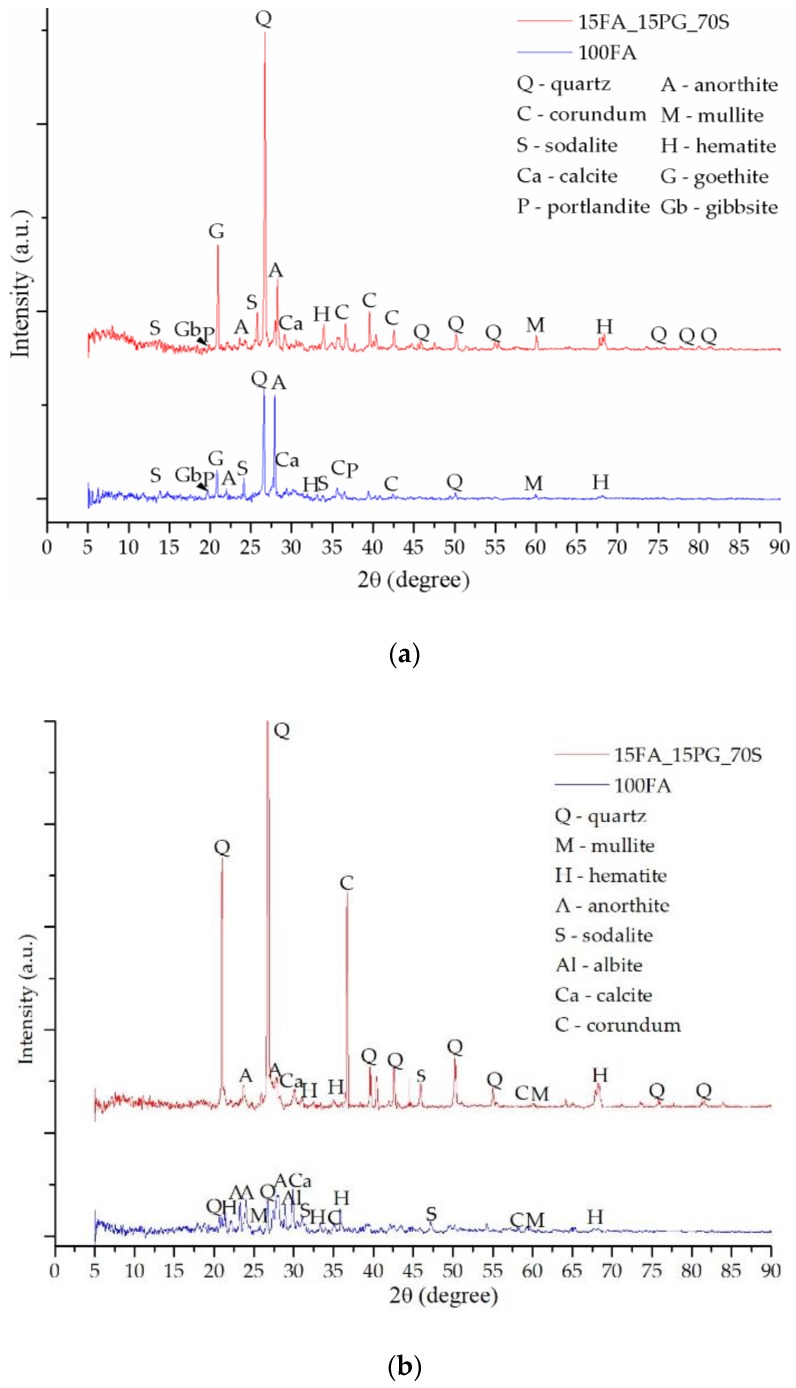
XRD patterns of sample 100FA and sample 15FA_15PG_70S: (**a**) prior TG-DTA analysis; (**b**) after TG-DTA analysis.

To confirm the portlandite and goethite decomposition during heating, XRD analysis has been performed after TG-DTA tests. Therefore, the heated samples diffractograms—Figure 8b, Figure 9b and Figure 10b—shows high peaks intensity modifications, especially for calcite and hematite, due to the chemical reactions (Equations (4) and (5)) follow the portlandite and goethite decomposition. Moreover, because the geopolymerization continues during heating, anorthite reacts with the Na^+^ cations, creating new phase albite with the highest intensity peak at 27.85°, 2θ.

These type of geopolymers have multiple phases which present similar XRD patterns, and, according to the database, one peak corresponded to multiple phases in this study we have presented, which was the phase with the highest intensity on the diffractograms. Therefore, after STA analysis, some peaks were changed to other phases because the phase intensity is different, e.g., the highest intensity peak at 21.09°, 2θ prior STA corresponds to goethite, yet, after STA it appears at 20.85°, 2θ and corresponds to quartz.

Following the replacement of 70% of the fly ash powder specific to the 100FA sample with sand particles, it was found that the intensity of some phases increased exponentially (Figure 9) while the specific phases of activation decrease significantly as a result of the reduction of the Al content available in the system.

The XRD diffractogram of the sample 15FA_15PG_70S (Figure 10b) shows a decrease in peaks intensity specific to the aluminum-containing compounds due to the sample ash content reduction.

Because the differences between the diffractograms specific to the raw material and those of the geopolymer samples are small, we can consider that the geopolymers have a granular structure whose surface is covered by phases resulting from the geopolymerization, which helps to bind them, resulting in a semi-crystalline structure.

The diffractogram of the thermal power plant fly ash shows mainly phases specific to the compounds of Al and Si, and at the trace level Fe, Ca, or Ti compounds can be observed. Following activation, an additional phase specific to zeolites appeared known as sodalite, while the initial phases showed minimal changes.

## 4. Conclusions

In the analyzed temperature range, the fly ash-based geopolymers exhibited high mass loss due to the removal of free and physically bound water molecules at up to 300 °C. Above this temperature multiple compounds, such as goethite, portlandite, gibbsite, etc. decomposed due to the OH groups (chemically bound water) removed. Yet, it was observed that the mass lose percentage depends on the sample fly ash content. Therefore, the hygroscopicity, as well as the concentration of unstable compounds, are strongly related to the matrix structure.

Taking into account the high number of chemical elements and so many possibilities of compounds formation during heating, such phase transition and structural modifications will result in geopolymers characteristics changing. Therefore, in order to obtain a much thermally stable geopolymer, based on indigenous fly ash, a high percentage of natural aggregates should be introduced in the matrix.

The glass powder introduction in the matrix will result in a denser sample, yet, due to the high calcium content, the thermal stability at high temperatures decreases.

## Figures and Tables

**Table 1:** Indigenous fly ash oxide chemical composition.

Oxide	SiO_2_	Al_2_O_3_	Fe_x_O_y_	CaO	K_2_O	MgO	TiO_2_	Na_2_O	P_2_O_5_	Oth ^1^
%, weight	47.80	28.60	10.20	6.40	2.40	2.00	1.30	0.60	0.40	0.30
Stat. error, %	0.32	0.27	0.95	0.77	0.71	1.09	1.81	0.63	0.24	-

^1^ Sum of chemical elements lower than 0.1%.

**Table 2:** Glass powder oxide chemical composition.

Oxide	SiO_2_	Al_2_O_3_	Fe_x_O_y_	CaO	MgO	Na_2_O	Oth ^1^
%, weight	70–71	1.5–2	0.8–1	9–11	2–3	12–14	<0.1

^1^ Sum of chemical elements lower than 0.1%.

**Table 3:** Samples components mix proportion.

Sample	Liquid Component, % Weight		Solid Component, % Weight		
	Na_2_SiO_3_	NaOH	Fly Ash	Glass Powder	Sand
100FA	60	40	100	0	0
70FA_30PG	60	40	70	30	0
30FA_70S	60	40	30	0	70
15FA_15PG_70S	60	40	15	15	70
